# Detection of lipoarabinomannan (LAM) in urine is indicative of disseminated TB with renal involvement in patients living with HIV and advanced immunodeficiency: evidence and implications

**DOI:** 10.1093/trstmh/trw008

**Published:** 2016-02-16

**Authors:** Stephen D. Lawn, Ankur Gupta-Wright

**Affiliations:** a Department of Clinical Research, Faculty of Infectious and Tropical Diseases, London School of Hygiene and Tropical Medicine, London, UK; b The Desmond Tutu HIV Centre, Institute of Infectious Disease and Molecular Medicine, Faculty of Health Sciences, University of Cape Town, Cape Town, South Africa; c Malawi-Liverpool-Wellcome Trust Clinical Research Program, University of Malawi College of Medicine, Blantyre, Malawi

**Keywords:** Diagnosis, Disseminated TB, HIV-associated TB, LAM, Lipoarabinomannan, Urine

## Abstract

TB is the leading cause of HIV/AIDS-related deaths globally. New diagnostic tools are urgently needed to avert deaths from undiagnosed HIV-associated TB. Although simple assays that detect lipoarabinomannan (LAM) in urine have been commercially available for years, their specific role and utility were initially misunderstood, such that they have been slower to emerge from the diagnostics pipeline than otherwise might have been expected. In this article, we review and explain how urine-LAM assays should be understood as diagnostics for disseminated TB in HIV-positive patients with advanced immunodeficiency, in whom haematogenous TB dissemination to the kidneys serves as the primary mechanism by which LAM enters the urine. These insights are critical for the appropriate design of studies to evaluate these assays and to understand how they might be most usefully implemented. This understanding also supports the 2015 WHO recommendations on the restricted use of these assays in sick HIV-positive patients with advanced immunodeficiency.

## Introduction

In this new era of the United Nations Sustainable Development Goals (SDGs), the international community has committed to ending the HIV/AIDS and TB epidemics by 2030.^1^ In 2014, the World Health Assembly adopted the WHO's post-2015 End TB Strategy, with targets to assess progress using data from 2015 as the baseline.^2^ Goals to be achieved by 2030 include an 80% reduction in new TB cases and a 90% reduction in TB deaths. Furthermore, achieving an incidence of <10 cases per 100 000 people per year was defined as being sufficient to ‘end’ the global TB epidemic.

The remaining task, however, is huge.^3^ WHO reported that in 2014 alone, 9.6 million people developed TB (12% HIV co-infected) and 1.4 million died from TB disease (43% HIV co-infected). Overall, TB remains the leading cause of HIV-related deaths globally. A meta-analysis of hospital post-mortem studies of HIV-related deaths included nine studies from across sub-Saharan Africa.^4^ These found that between 32 and 67% (pooled summary estimate, 43.2%; 95%CI 38.0–48.3) of HIV-positive adult cadavers had evidence of TB at autopsy, and that in over 90% of cases it was deemed the primary cause of death.^4^ Disease was disseminated in 87.9% of TB cases, and remained undiagnosed at death in nearly half of cases (45.8%). These data highlight the critical need to improve the ascertainment of disseminated HIV-associated TB. To achieve its ambitious goals, the WHO End TB strategy has outlined the requirement for ‘intensified research and innovation’,^1^ including the development and implementation of new, simple and accurate diagnostic assays for TB.

For many years, the development of point-of-care assays has been foremost on the TB research agenda. One such assay methodology for TB diagnosis to emerge over the past 20 years has been the detection of the mycobacterial cell well glycolipid antigen lipoarabinomannan (LAM) in urine using immune-capture assays. Much of the pioneering work on this was led by Prof. Stefan Svenson at the Karolinska Institute in Sweden, who demonstrated the proof of concept of urine-LAM detection as a means for TB diagnosis.^5^ This early methodology required complex sample processing, which was beyond the capacity of most laboratories in high TB burden countries, and this was viewed as an insurmountable obstacle to implementation. However, an ELISA that required only minimal urine processing was developed and marketed just over 10 years ago. The results of the first assessment of its diagnostic accuracy in Tanzania were very encouraging,^6^ with high specificity among asymptomatic volunteers and high sensitivity in patients with laboratory-confirmed pulmonary TB. Moreover, diagnostic accuracy was similar in HIV-positive and HIV-negative patients. Had these findings been replicated in larger studies in different settings, this assay would have constituted a substantial breakthrough.

Optimism regarding this assay quickly evaporated, however, as many subsequent studies reported poor diagnostic accuracy. A meta-analysis in 2011^7^ concluded these assays had “suboptimal sensitivity for routine clinical use’ and many dismissed urine-LAM detection as a viable diagnostic methodology. In retrospect, many of the earlier studies targeted non-ideal patient groups (HIV-negative patients or HIV-positive patients with high CD4 counts). However, progress came when the assay was applied to HIV-positive adults with advanced immunodeficiency,^8^ in whom specificity was high and sensitivity, although only moderate at best, was highest among the sickest, most vulnerable patients in whom slow culture-based diagnosis was otherwise often required. The next breakthrough came with the development of a 30-minute lateral-flow assay (strip-test) version of the assay marketed as ‘Determine TB-LAM’ (Alere Inc. Waltham, MA, USA). This assay format required neither laboratory infrastructure nor equipment, and was far lower cost per test than the Xpert MTB/RIF assay (Cepheid, Sunnyvale, CA, USA). The results of the ELISA and lateral flow assay formats were remarkably similar, both showing markedly much greater sensitivity among those patients with the lowest CD4 counts.^9^ Thus, urine LAM detection emerged as a means for rapid diagnosis of TB in people living with HIV and advanced immunodeficiency.

Urine-LAM immune-capture assays have not been embraced by the HIV and TB communities as fully or rapidly as one might have expected. We propose that this is not only due to unfavourable data arising from earlier studies in which the assay was evaluated in inappropriate or non-ideal patient groups and using sub-optimal study methodologies. We believe that, in addition to these factors, the fundamental basis of the assays had not been correctly understood—in particular, the mechanism by which LAM enters urine. At first, this might seem an esoteric, academic issue, but we highlight why this issue is of fundamental importance for the appropriate understanding and application of these assays.

Early literature concerning urine-LAM assays presumed that LAM-antigenuria resulted from simple renal filtration of free circulating LAM in the bloodstream.^6,10^ This notion seemed plausible since it was known that LAM was produced in large quantities by *Mycobacterium tuberculosis* (MTB) at sites of disease from which it could enter the blood-stream.^11^ Moreover, as a 17 kDa glycolipid (similar in size to myoglobin) it should readily traverse the renal glomerular basement membrane. Once produced at sites of TB, whether pulmonary or extrapulmonary, it was known that LAM was detectable in blood, albeit following extensive sample processing in view of the high degree of immune-complexing with host anti-LAM antibodies.^12^ Renal filtration of circulating LAM also plausibly explained why the assay detected both pulmonary and extrapulmonary forms of TB. However, here we present a range of direct and indirect evidence that collectively indicates that LAM antigenuria results from renal involvement with TB and not from simple renal filtration of LAM from the bloodstream into urine (Table 1).

**Table 1.** Direct and indirect evidence that urine lipoarabinomannan (LAM) antigenuria is due to renal involvement with haematogenously disseminated TB, and not free filtration of LAM into the urine

Observation	Evidence	References
LAM is unlikely to be able to pass from systemic circulation into the urine via the renal glomerulus	LAM in the bloodstream is mostly immune-complexed or bound to high-density lipoprotein These LAM containing molecules are too large to freely filter in the renal glomerulus	Sada et al. 1992^12^
		Sakamuri et al. 2013^13^
		Haraldsson et al. 2008^14^
LAM concentration in the urine does not increase during the first weeks of TB treatment	Serum LAM concentration likely increases early after the massive mycobactericidal effect of anti-TB treatment.	Wood et al. 2012^15^
	If LAM was freely filtered in the kidneys, LAM concentration in the urine would also be expected to increase early after treatment	Bekker et al. 1998^45^
Most LAM-positive urines test XpertMTB/RIF-positive	Xpert MTB/RIF detects *M. tuberculosis* DNA in whole bacilli (not free DNA), suggesting most LAM-positive urines contain whole *M. tuberculosis* bacilli	Wood et al. 2012^15^
		Lawn et al. 2012^21^
		Blakemore et al. 2010^19^
Frequent LAM-positive urine in patients with *M. tuberculosis* bacteraemia	The strong association between *M. tuberculosis* bacteraemia and LAM-positive urine is very plausibly linked mechanistically by haematogenously disseminated renal TB	Manabe et al. 2014^28^
		Nakiyingi et al. 2015^29^
		Nakiyingi et al. 2014^30^
		Lawn & Kerkhoff 2015^31^
In patients with disseminated TB at autopsy, renal TB is common	Prevalence of renal TB is similar to the sensitivity of LAM-positive urine in this population	Lanjewar et al. 2011^25^
		Ansari et al. 2002^24^
		Cox et al. 2015^17^
Post-mortem renal TB in HV-positive patients is associated with LAM-positive urine	A Ugandan Autopsy study revealed frequent renal TB in those whose urine also tested LAM-positive and all LAM-positive patients had haematogenously disseminated TB No patients with renal TB were urine LAM-negative	Cox et al. 2015^17^

## Simple filtration of LAM at the renal glomerulus is implausible

In addition to being immune-complexed within blood by anti-LAM antibodies, LAM in the bloodstream is also incorporated within high density lipoprotein (HDL) particles.^13^ Both immune-complexed or HDL-associated LAM would be unable to pass from the systemic circulation through the intact basement membrane in the renal glomerulus.^14^ Whilst glomerular dysfunction might hypothetically allow LAM-containing-complexes to pass into the urine, clinical studies have not found associations between urine-LAM detection and proteinuria (a marker for glomerular damage),^15,16^ and there is no histological evidence of glomerular damage at autopsy in patients who were urine-LAM positive.^17^

LAM is released in large quantities by actively replicating or dying mycobacteria, serving as an immune-modulating virulence factor. If LAM were freely filtered into urine, it would be expected that during early TB therapy when massive mycobacterial killing occurs, urine concentrations of LAM would rise in parallel with blood concentrations. However, the fact that urine-LAM levels do not increase during early TB treatment is suggestive of a mechanism other than simple renal filtration.^15^

## Urine microbiology supports the renal TB hypothesis (Table 1)

Urine culture has previously been shown to provide a high diagnostic yield in an American cohort of patients with AIDS and extrapulmonary TB in whom 77% tested urine-culture- positive.^18^ If the presence of LAM in the urine of HIV-infected patients were the result of renal TB, one would expect that *M. tuberculosis* would be culturable from LAM-positive urine samples. However, data correlating urine LAM status and urine culture status are unfortunately lacking, at present. Nevertheless, other important evidence comes from testing of urine samples with the Xpert MTB/RIF assay. The Xpert MTB/RIF assay detects the presence of whole MTB bacilli through amplification of organism-associated DNA (rather than detection of free DNA).^19,20^ Studies testing the urine of HIV-TB patients using TB-LAM and Xpert MTB/RIF have shown a large overlap between the assays, with ≥50% of LAM-positive urine samples also testing positive by Xpert MTB/RIF.^21–23^ The detection of whole MTB bacilli in urine of these LAM-positive patients strongly corroborates the notion of renal TB as the source of urinary-LAM.

## Evidence of renal TB from post-mortem studies

Renal TB in the form of microabscesses in this patient population arises as a result of haematogenous dissemination and is a common autopsy finding in patients who have died from disseminated HIV-associated TB (50–69%).^24–27^ In patients with advanced HIV-infection (typically CD4 cell counts of <100 cells/mm^3^) a strong association between MTB bacteraemia and urine LAM-positivity has been observed, with 70–90% of bacteraemic patients also having detectable LAM-antigenuria.^28–31^ Renal TB readily provides a mechanism for this association.

The strongest evidence explaining the mechanism of urine LAM-antigenuria is provided by post-mortem studies. Cox et al. demonstrated histological evidence of renal TB in the majority of deceased patients with LAM detected in their urine.^17^ All LAM-positive patients had evidence of either renal or disseminated TB. Renal TB is also frequently found in cadavers of adults with disseminated TB at post-mortem, typically being seen in more than 50% of such cases.^24–27^

That LAM-antigenuria is due to renal involvement with haematogenously disseminated TB rather than filtration of LAM also explains several observations about urine-LAM assays, with implications for future research.

## What are the implications?

Thus far, we have presented evidence supporting the notion that LAM-antigenuria results from renal involvement with TB arising from haematogenous TB dissemination and not from simple renal filtration of circulating LAM. This understanding is critical as it provides many important insights into the urine-LAM assays and their particular niche within the TB diagnostics armamentarium. Here we now discuss the implications of this knowledge (Tables 2 and 3).

**Table 2.** Observations about urine lipoarabinomannan (LAM) assays explained by LAM-antigenuria resulting from renal involvement with TB

Observation	Evidence	References
The utility of the assay is almost entirely restricted to testing adult patients who are HIV-infected.	Haematogenously disseminated TB, and therefore renal TB, is rare amongst immune-competent patients If LAM were freely filtered sensitivity amongst pulmonary TB cases should be higher	Minion et al. 2011^7^
Assay sensitivity is very strongly associated with blood CD4 cell count, (highest among CD4 counts <50 cells/μL and lowest among CD4 counts >200 cells/μL).	Haematogenously disseminated TB, and therefore renal TB, is more common with more advanced immunosuppression in HIV	Lawn et al. 2009^8^
		Lawn et al. 2012^9^
		Minion et al. 2011^7^
The assay has no utility among children	Paediatric TB is usually paucibacillary with rare dissemination of TB disease, even in the context of HIV	Nicol M et al. 2014^33^
Urine LAM assays should not be specifically targeted to the investigation of HIV-positive patients with respiratory symptoms of TB	Diagnostic yield of LAM is unrelated to respiratory symptoms (cough or sputum production)	Lawn et al. 2014^23^
LAM-antigenuria is a strong independent predictor of mortality among patients with HIV-associated TB.	Renal and haematogenously disseminated TB is associated with greater mycobacterial burden, which is likely to adversely impact prognosis	Lawn et al. 2014^35^
		Talbot et al. 2012^37^
		Drain et al. 2015^38^
Studies of urine-LAM find much higher specificity when the reference standard for TB diagnosis includes testing of extra-pulmonary samples in addition to sputum	Testing extra-pulmonary samples increases the chances that disseminated TB is correctly detected, and fewer urine-LAM positive patients will be misclassified as ‘not TB’ due to inadequate reference standards.	Cox et al. 2015^17^
		Lawn et al. 2014^35^
		Nakiyingi et al. 2014^30^

**Table 3.** Implications of lipoarabinomannan (LAM) antigenuria being a consequence of disseminated renal

Issue	Implications	References
Targeting the assay to appropriate patient groups	LAM assays should be prioritised for testing sick HIV-positive adults, such as those requiring acute hospital admission rather than ambulatory out-patients Interpretation of urine-LAM results requires knowledge of HIV status There are no data showing utility for testing in children	Nakiyingi et al. 2014^30^
Design of diagnostic accuracy studies	The reference standard for disseminated TB requires testing more than one specimen type, a single sputum culture is inadequate Ideally this would include mycobacterial blood cultures and/or urine Xpert MTB/RIF assay testing	Lawn et al. 2015^41^
Design of studies to assess incremental diagnostic yield of urine-LAM testing	Should include patients both with and without respiratory symptoms	Lawn et al. 2014^46^
Studies of clinical impact of Urine-LAM testing	Studies should focus on acute HIV-positive medical admissions in whom undiagnosed TB and mycobacteraemia is common. e.g. LAMRCT and STAMP trials^42,43^	Gupta et al. 2015^4^
		Lawn & Kerkhoff 2015^31^
Patients testing LAM-positive have poorer prognosis than those testing LAM-negative	Patients testing urine-LAM-positive are much sicker and have poorer prognostic characteristics compared with those with TB who testing LAM-negative	Manabe et al. 2014^28^
		Lawn et al. 2013^36^

## Renal TB explains why urine-LAM assays only have utility in HIV-infected patients

Diagnostic accuracy studies have found the utility of urine-LAM assays to be almost entirely restricted to HIV-positive adults with advanced immunodeficiency.^7^ Disseminated TB, and therefore renal TB, are comparatively much less common among immune-competent patients. Furthermore, assay sensitivity is very strongly associated with low CD4 cell counts, being highest among those with CD4 counts <50 cells/μL,^7,32^ the very patient group with highest risks of mycobacteraemia and disseminated and renal TB. Thus, understanding the mechanism of LAM-antigenuria directly explains why urine LAM-assays have utility restricted to a defined patient group and why the earlier diagnostic accuracy studies focusing on other patient groups generated largely unfavourable data. The results of studies of urine-LAM assays were also disappointing in children with no useful diagnostic accuracy observed, which is likely to be explained by the paucibacillary nature of paediatric TB, contrasting with the overwhelming multi-bacillary nature of disseminated TB in adults with advanced HIV.^4,33^

## Higher mortality in urine-LAM positive patients

Several studies have now demonstrated that HIV-TB co-infected patients who test urine-LAM-positive have much poorer prognostic characteristics and higher mortality risk compared to cases testing LAM-negative.^28,34–36^ This increased mortality risk persists even after adjustment for important risk factors such as CD4 cell count and anaemia.^35,37,38^ The fact that urine LAM-positivity serves as a marker of disseminated/renal TB, is an entirely plausible reason for this association with prognosis, as HIV-TB patients with mycobacteraemia are known to have high mortality risk.^39,40^ Diagnosing mycobacteraemia by liquid culture of blood samples can take 2 to 6 weeks, and is largely unavailable outside research facilities in most high burden settings. However, urine-LAM testing can quickly (a 30 min test) identify 80% of patients with mycobacteraemia,^31^ therefore expediting the diagnosis of MTB bacteraemia among patients with high mortality risk. Further research is required to see if interventions in addition to expediting TB treatment can reduce this very high mortality risk.

## Underestimation of urine-LAM specificity due to an inadequate reference standard for disseminated TB in diagnostic accuracy studies

Finally, understanding that urine LAM-positivity represents disseminated and renal TB helps to explain why assessments of Determine TB-LAM specificity are sub-optimal in many studies in which reference standard testing for TB was restricted to a single respiratory sample.^41^ LAM-positive disseminated and renal TB may be very easily missed by such a reference standard, especially among very sick patients unable to produce good quality sputum samples and would, therefore, be inappropriately misclassified as ‘TB-free’. Specificity of urine LAM-detection is ≥99% when the reference standard includes comprehensive extra-pulmonary sampling that is much more likely to detect disseminated TB and thereby accurately classifies patients as having TB or being TB-free.^17,23,41^

## Conclusion

The advent of a simple ELISA-based format for urine-LAM detection for TB diagnosis was greeted with much optimism in 2005, and yet many were perplexed by the results of subsequent studies, which were designed based on a limited understanding of these assays, including the mechanism of LAM-antigenuria, which has profound implications. In effect, the advent of urine-LAM assays heralded the development of simple assays for rapid diagnosis of disseminated HIV-associated TB with renal involvement, providing not only rapid diagnosis but also key prognostic information. This emerging understanding has now culminated in randomised trials of urine-based diagnosis to determine whether their addition to routine diagnostic screening algorithms averts deaths from HIV-associated TB by improving and accelerating the TB diagnostic yield.^42,43^ Results of such studies are awaited.

This paper highlights the critical importance that new diagnostic assays are thoroughly understood mechanistically and are applied to the most appropriate patient groups. Without this, there is a risk that some of the output of the TB diagnostics developmental pipeline might potentially be squandered. However, the mechanistic framework for understanding the utility of the urine LAM assays that we have described in this article is strongly supportive of the WHO recommendations regarding the use of these assays for diagnosis. These specifically restrict or target the use in HIV-positive patients who have the highest risk of disseminated TB (notably in-patients who are sick and have CD4 cell counts <100 cells/µL).^44^
